# Rotavirus Rearranged Genomic RNA Segments Are Preferentially Packaged into Viruses Despite Not Conferring Selective Growth Advantage to Viruses

**DOI:** 10.1371/journal.pone.0020080

**Published:** 2011-05-17

**Authors:** Cécile Troupin, Aurélie Schnuriger, Sarah Duponchel, Claire Deback, Nathalie Schnepf, Axelle Dehee, Antoine Garbarg-Chenon

**Affiliations:** 1 Micro-Organismes, Molécules Bioactives et Physiopathologie Intestinale, Université Pierre et Marie Curie - Paris 6, Paris, France; 2 ERL U1057/UMR 7203, Institut National de la Santé et de la Recherche Médicale, Paris, France; 3 Laboratoire de Virologie, Hôpital Armand Trousseau, Assistance Publique - Hôpitaux de Paris, Paris, France; Nanyang Technological University, Singapore

## Abstract

The rotavirus (RV) genome consists of 11 double-stranded RNA segments. Sometimes, partial sequence duplication of an RNA segment leads to a rearranged RNA segment. To specify the impact of rearrangement, the replication efficiencies of human RV with rearranged segments 7, 11 or both were compared to these of the homologous human wild-type RV (wt-RV) and of the bovine wt-RV strain RF. As judged by viral growth curves, rotaviruses with a rearranged genome (r-RV) had no selective growth advantage over the homologous wt-RV. In contrast, r-RV were selected over wt-RV during competitive experiments (i.e mixed infections between r-RV and wt-RV followed by serial passages in cell culture). Moreover, when competitive experiments were performed between a human r-RV and the bovine wt-RV strain RF, which had a clear growth advantage, rearranged segments 7, 11 or both always segregated in viral progenies even when performing mixed infections at an MOI ratio of 1 r-RV to 100 wt-RV. Lastly, bovine reassortant viruses that had inherited a rearranged segment 7 from human r-RV were generated. Although substitution of wt by rearranged segment 7 did not result in any growth advantage, the rearranged segment was selected in the viral progenies resulting from mixed infections by bovine reassortant r-RV and wt-RV, even for an MOI ratio of 1 r-RV to 10^7^ wt-RV. Lack of selective growth advantage of r-RV over wt-RV in cell culture suggests a mechanism of preferential packaging of the rearranged segments over their standard counterparts in the viral progeny.

## Introduction

Group A rotaviruses (RV), members of the *Reoviridae* family, are a major cause of infantile viral gastroenteritis and are responsible for approximately 600 000 deaths each year [1], [2]. The RV genome consists of 11 segments of double-stranded RNA (dsRNA), which can be separated by polyacrylamide gel electrophoresis (PAGE). dsRNA profiles (electropherotypes) of wild-type RV (wt-RV) typically show four size classes of segments according to their molecular weight. Variations in the mobility of individual RNA segments allow a genetic characterization of RV strains. Some RV strains show unusual dsRNA profiles, in which standard size segments are replaced by rearranged forms of larger size (for a review see [3], [4]). Such rotaviruses with a rearranged genome (r-RV) were first isolated from chronically infected immunodeficient children [5], [6] and animals [7]–[9]. Gene rearrangements can also be generated in cell culture by serial passages of RV at high multiplicity of infection (MOI) [10]–[14].

Gene rearrangements mostly involve segment 11, which encodes the two nonstructural proteins NSP5 and NSP6, and less frequently segments 5 to 10. In most cases, the rearrangement results from a partial head-to-tail duplication of the gene: the sequence includes an unmodified 5′ untranslated region (UTR) and open reading frame (ORF) followed by a duplication of the 5′-region, which starts from various positions after the stop codon and extends up to the 3′ end, leading to a long 3′ UTR [3]. Thus, rearranged genes usually express unmodified proteins [13], [15], [16], unless when rearrangement is associated with point mutations in the ORF, as reported for a gene 6 rearrangement that affects the VP6 protein stability [17]. Less frequently, sequence duplication may occur within the ORF. Gene rearrangements leading to a modified ORF have been described for segments 5 [18], [19] and 7 [16]. In both cases, the resulting r-RV have retained their capacity to grow in cell culture, although expressing a modified NSP1 or NSP3 protein.

In a previous study, we have shown that a minority of copies of rearranged forms of segment 11 can be produced along with a majority of wt viral genomes, in the course of acute RV infection of immunocompetent children [20]. Nevertheless, r-RV have only seldom been found to circulate among immunocompetent children [20]–[26]. This might be due to a growth advantage of wt-RV over r-RV and/or to the short duration of acute infection, leaving no time for r-RV to emerge. In contrast, during chronic infection of immunodeficient children, r-RV usually overgrow wt-RV in the viral progeny [5], [6], [15], [16], [27], [28], indicating a possible selective advantage of r-RV over wt-RV when impairment of the immune response allows the virus to undertake many replication cycles over a long period of time.

Only a few studies have attempted to assess the effect of gene rearrangement on viral replication. It has been reported that selection of r-RV over wt-RV can occur during serial passages in cell culture [11], [29]–[31], although it may depend on the MOI [11]. However, it is unknown whether selection of r-RV over wt-RV is due to a selective growth advantage of the virus or to a selective packaging advantage of the rearranged segments. Recently, we have reported a reverse genetics system for RV based on the ability of rearranged segments to be maintained and amplified in the viral progeny without the need of any selective pressure other than selection inherent to cell culture [32]. This system has allowed recovering engineered mono-reassortant infectious RV having incorporated an exogenous *in vitro* modified cDNA-derived rearranged segment 7.

The present study aims to specify whether rearranged segments confer a selective growth advantage to the virus or have a selective advantage for being packaged into the virus. For this purpose, the replication efficiencies of previously described human r-RV with rearranged segments 7, 11 or both [16], were compared to these of the homologous human wt-RV and of the bovine wt-RV strain RF. Lack of selective growth advantage of r-RV over wt-RV in cell culture suggests a mechanism of preferential packaging of the rearranged segments over their standard counterparts in the viral progeny.

## Materials and Methods

### Viruses and cells

**Figure 1:**
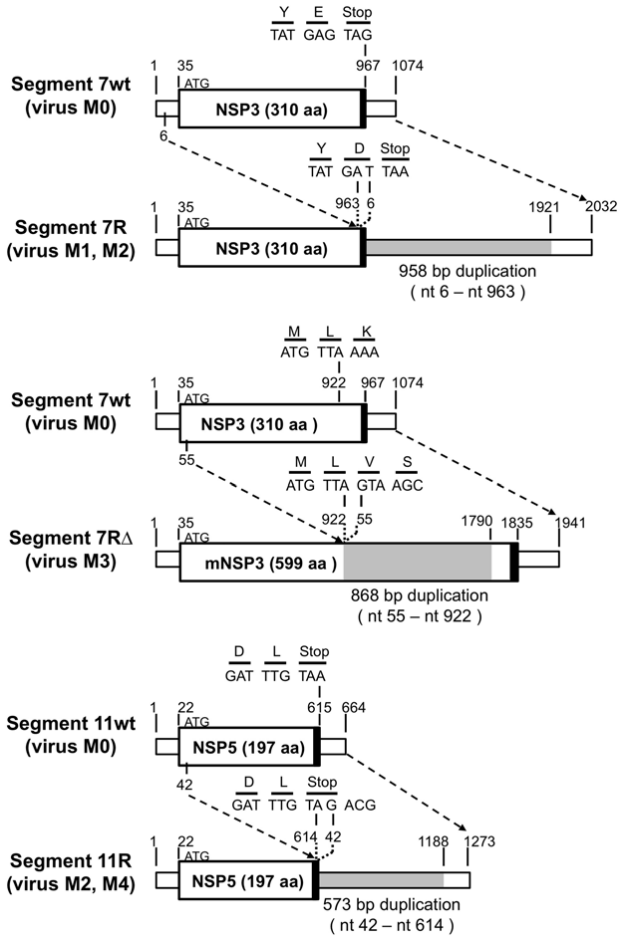
Schematic diagram of human wt and rearranged segments 7 and 11. For each segment, the ORFs are indicated by large boxes and the UTRs by small boxes. Thick lines indicate the stop codons. The duplicated sequence in each of the rearranged segments is shaded in grey. The nucleotides (nt) and their translations (single letter amino-acid code) implicated in rearrangement are detailed above each segment. Numbers refer to nt. Segment 7R (virus M1 and M2) contains a 958 bp sequence duplication (corresponding to nt 6 to nt 963) inserted at position 963 of the wt sequence; segment 7RΔ (virus M3) contains a 868 bp sequence duplication (nt 6 to nt 922) inserted at position 922 of the wt sequence and results in a modification of the NSP3 ORF; segment 11R (virus M2 and M4) contains a 573 bp sequence duplication (nt 42 to nt 614) inserted at position 614 of the wt sequence.

Viruses M0, M1, M2 and M3 are four previously described cell-culture adapted viral clones, which all derived from the same human rotavirus clinical isolate M isolated from the stool of a chronically infected child with severe combined immunodeficiency syndrome [16]. Virus M0 has 11 standard dsRNA segments, virus M1 has a rearranged segment 7, termed 7R (Genbank AF338247), virus M2 harbors the rearranged segment 7R and a rearranged segment 11, termed 11R (Genbank AF338245), and virus M3 a rearranged segment 7, termed 7RΔ (Genbank AF338248) (see figure 1 for details). The coding sequences of segments 7R and 11R are identical to those of the wt segments 7 and 11 of virus M0.). Segment 7R contains a full repetition of the NSP3 ORF. However, virus M1 does not over-express the NSP3 protein as judged by Western blot analysis, suggesting that the duplicated second ORF is most probably untranslated [16]. Segment 7RΔ has a modified ORF encoding an almost full-length duplicated NSP3 protein (mNSP3). Human rotavirus M4 carrying the rearranged segment 11R and a standard segment 7 is a reassortant virus that was obtained by mixed infection with M0 and M2 followed by three plaque-to-plaque cloning steps in MA-104 cells. Thus, viruses M0 to M4 share the same genetic background. The bovine RV strain RF was used as a reference strain of different genetic background. The same clonal stocks of human strains M0 (1.65×10^7^ PFU/ml), M1 (2.50×10^6^ PFU/ml), M2 (2.09×10^7^ PFU/ml), M3 (2.78×10^7^ PFU/ml), M4 (8.22×10^6^ PFU/ml), or bovine strain RF (5.50×0^8^ PFU/ml) were used in all experiments. MA-104 cell line was cultured in Dulbecco's minimum essential medium (DMEM) supplemented with 10% foetal calf serum, 2 mM glutamine, 100 U/ml penicillin, and 1 µg/ml streptomycin. RV propagation on confluent monolayers of MA-104 cells, and plaque assays for virus titration and cloning were performed as previously described [16], [32].

### Virus growth curves

For each virus, confluent MA-104 cells grown on 25 cm^2^ flasks were inoculated at the same MOI of 0.5 PFU/cell. The infected cell cultures were incubated at 37°C and harvested at 2, 6, 8, 10, and 18 hours post-infection. Infected cell cultures were freeze-thawed three times, centrifuged at low speed to remove cell debris, and aliquots of supernatants were kept frozen at −80°C. Virus titers were determined by plaque assay in MA-104 cells.

### Co-infection experiments

Confluent monolayers of MA-104 cells in 25 cm^2^ flasks were co-infected by a mixture of two RV, one with a rearranged genome (r-RV) and the other with a wild-type genome (wt-RV). Unless otherwise stated, mixed infections were performed using several MOI ratios of r-RV to wt-RV; 1∶1 (at an MOI of 0.3 PFU/cell for each virus); 1∶20 (1.5×10^−2^ and 0.3 PFU/cell for r-RV and wt-RV, respectively); 1∶100 (0.3×10^−2^ and 0.3 PFU/cell for r-RV and wt-RV, respectively) in a final volume of 500 µL. The resulting culture was serially propagated on MA-104 cells in 25 cm^2^ flasks for 48 h (using 1∶8 of the undiluted cell culture lysate as inoculum), and at each passage aliquots were kept frozen at −80°C for further analysis. As co-infection experiments followed by serial passages in cell culture were performed in order to evaluate whether competition between viruses occurs, such experiments are further designated as “competitive experiments”.

### Nucleic acid analysis

For PAGE and RT-PCR analysis, RV genomic dsRNA was extracted from cell culture aliquots, using RNA PLUS (Bioprobe System) or Tri-Reagent LS (Euromedex) according to manufacturer's recommendations. RNA genomic profiles were determined by PAGE in 14% polyacrylamide gels for 16 h at 200 V at room temperature followed by ethidium bromide staining. The RT-PCR assay for specific detection of rearranged segments 7 in the viral progeny was performed as previously described [32]. The RT-PCR assay had a sensitivity threshold ratio of one rearranged segment 7 to 10^5^ wt segments 7.

### Protein Analysis

To detect NSP3 viral protein, MA-104 cell cultures were harvested 18 h post infection in a lysis buffer containing 50 mM Tris-HCl (pH 6.8), 2% sodium dodecyl sulfate (SDS), and 2% ß-mercaptoethanol. SDS-PAGE and Western blotting were performed as previously described [16], using the monoclonal mouse anti-NSP3 ID3 antibody [33] kindly provided by Didier Poncet.

## Results

### Rearranged segments do not confer any selective growth advantage to viruses sharing the same genetic background

**Figure 2:**
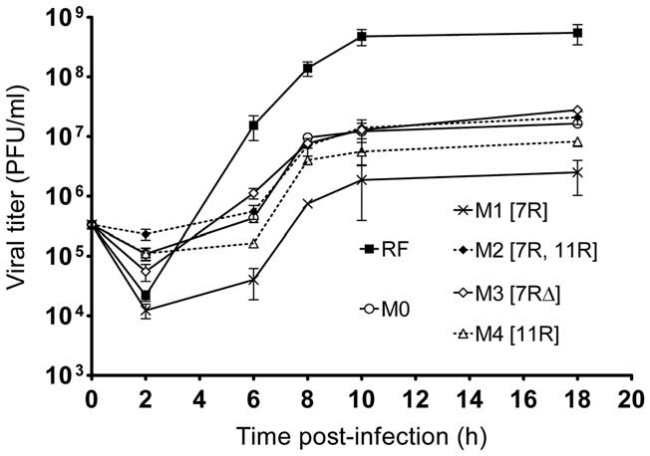
One-step viral growth curves of human wt-RV and r-RV. For each virus, confluent MA-104 cells were inoculated at the same MOI of 0.5 PFU/cell. The infected cell cultures were harvested at 2, 6, 8, 10, and 18 hours post-infection and virus titers were determined by plaque assay in MA-104 cells. Human wt-RV M0 and r-RV M1 to M4 share the same genetic background. The rearranged segments of r-RV M1 to M4 are indicated in brackets. The bovine RV strain RF was used as control.

In order to compare growth kinetics during one replication cycle, viral growth curves of human wt-RV M0 and of r-RV M1 to M4, which share the same genetic background, were established in MA-104 cell culture by measuring viral titers over an 18 hours period of infection with the use of a plaque assay. Virus M0 has 11 standard dsRNA segments, virus M1 has a rearranged segment 7, termed 7R, virus M2 harbors the rearranged segment 7R and a rearranged segment 11, termed 11R, virus M3 has a rearranged segment 7, termed 7RΔ and virus M4 harbors the rearranged segment 11R (see figure 1 for details). The coding sequences of segments 7R and 11R are identical to those of the wt segments 7 and 11 of virus M0, while segment 7RΔ has a modified ORF encoding an almost full-length duplicated NSP3 protein (mNSP3) [16]. As shown in figure 2, growth kinetics of the wt-RV M0 and of r-RV M1 to M4 were similar. The wt-RV M0 and r-RV M2, M3 and M4 grew to titers of the same order, while r-RV M1 grew to titers 10 fold lower. Thus, human r-RV with rearranged segments 7R, 7RΔ and/or 11R replicated less than or equally to their wt-RV counterpart in MA-104 cell culture. The wt-RV M0 replicated slower and grew to titers 100 fold lower than the bovine RV strain RF, as usually reported for other human RV. Taken together these results indicated that rearranged segments do not confer any selective growth advantage to viruses sharing the same genetic background.

### r-RV are selected over wt-RV sharing the same genetic background

**Figure 3:**
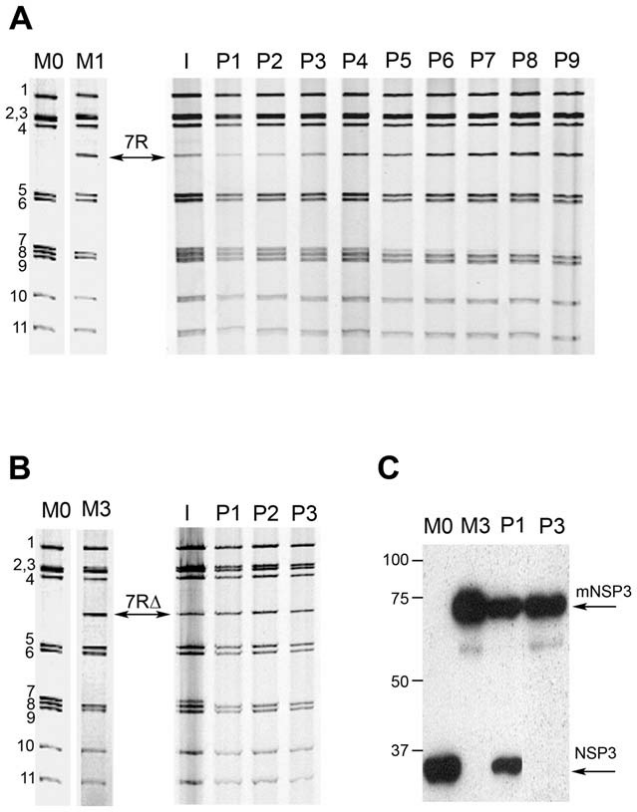
Competitive experiments between human wt-RV M0 and r-RV M1 or M3. Competitive experiments were performed by co-infecting MA-104 cells by wt-RV and r-RV at an MOI ratio of 1∶1 (0.3 PFU/cell for each virus). The resulting cell culture lysates were serially propagated in MA-104 cells. RNA profiles of viral progenies resulting from mixed infections by wt-RV M0 and r-RV M1 (A) or M3 (B) are shown. I and Pn indicate the initial inoculum used for mixed infections and the passage number, respectively. Numbers indicate the location of RNA segments. Arrows indicate the rearranged segment 7R from M1 and 7RΔ from M3. (C) Western-blot detection of the NSP3 protein expressed by the viral progenies resulting from the M0+M3 co-infection at passage 1(P1) and 3 (P3). Arrows indicate the NSP3 and the modified mNSP3 proteins encoded by wt segment 7 (M0 virus) and rearranged segment 7RΔ (M3 virus), respectively. Numbers indicate molecular size, in kilodaltons.

**Figure 4:**
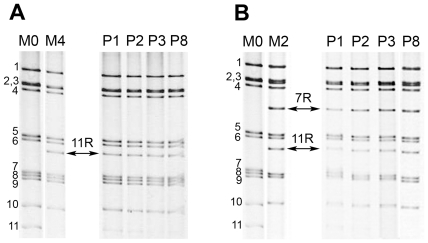
Competitive experiments between human wt-RV M0 and r-RV M4 or M2. Competitive experiments were performed as described in figure 3. RNA profiles of viral progenies resulting from mixed infections by wt-RV M0 and r-RV M4 (A) or M2 (B). Pn indicates the passage number. Numbers indicate the location of RNA segments. Arrows indicate rearranged segment 11R (M4 and M2 viruses) and 7R (M2).

In order to compare replication efficiency, competitive experiments were performed between r-RV and the homologous wt-RV. For this purpose, MA-104 cells were co-infected at the same MOI (0.3 PFU/cell) by wt-RV M0 and either of the r-RV M1-M4. Viral progenies resulting from mixed infections were serially propagated in MA-104 cells and at each passage the RNA profiles of the viral populations were analyzed by PAGE. Competitive experiments between wt-RV M0 and r-RV M1 resulted in the selection of viruses with an M1 RNA profile (Figure 3A). In the course of serial passages, RNA profiles showed a progressive increase in rearranged segment 7R intensity, along with a decrease in wt segment 7 intensity, which actually became undetectable at passage 9, indicating that segment 7R had replaced its wt counterpart in the viral progeny. Competition between wt-RV M0 and r-RV M3 resulted in the selection of viruses with an M3 RNA profile, with wt segment 7 being substituted by segment 7RΔ after only 3 passages (Figure 3B). This substitution was confirmed by Western-blotting using a monoclonal anti-NSP3 antibody: while both the wt NSP3 protein (encoded by segment 7) and the modified NSP3 protein mNSP3 (encoded by segment 7RΔ) were detected at the first passage, only mNSP3 was detected at passage 3 (Figure 3C). Similar results were obtained from competitive experiments between wt-RV M0 and r-RV carrying the rearranged segment 11R. Indeed, competition between M0 and M4 resulted in the selection of viruses with an M4 RNA profile, segment 11R replacing wt segment 11 after 8 passages (Figure 4A). Furthermore, competition between M0 and M2, which carries both rearranged segments 7R and 11R, led to the selection of viruses of the M2 type, with segments 7R and 11R replacing their wt counterparts after 8 passages (Figure 4B). The selection of r-RV in the viral progeny was also observed when the initial mixed infections were performed with a 1∶20 MOI ratio of r-RV M1 or M3 to wt-RV M0, although substitution of wt by rearranged segments required more passages to occur. These results indicated that r-RV were always selected in the viral progenies resulting from competitive experiments with wt-RV. However, considering that r-RV (M1 to M4) replicated less than or equally to the wt-RV M0, though sharing a same genetic background, it was unlikely that r-RV could have overgrown wt-RV, especially when mixed-infections were performed at MOI ratios favoring wt-RV. This rather suggested a preferential segregation of rearranged over wt segments in the viral progeny produced throughout cell passages.

### Rearranged segments preferentially segregate in the viral progeny resulting from mixed infections between r-RV and wt-RV of different genetic backgrounds

**Figure 5:**
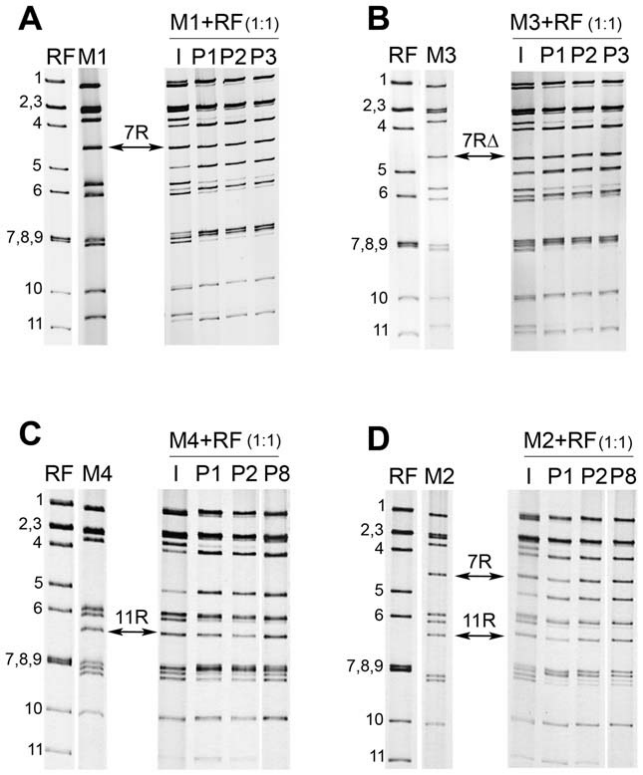
Competitive experiments between bovine wt-RV and human r-RV. Competitive experiments were performed by co-infecting MA-104 cells by the bovine wt-RV RF and one of the human r-RV at an MOI ratio of 1∶1 (0.3 PFU/cell for each virus). The resulting cell culture lysates were serially propagated in MA-104 cells. RNA profiles of viral progenies resulting from mixed infections by wt-bovine RF virus and human r-RV M1 (A), M3 (B), M4 (C), or M2 (D). RNA profiles of wt-bovine RF and human r-RV show differences of mobility for 8 RNA segments (segments 1, 4–6, 8–11). I and Pn indicate the initial inoculum used for mixed infections and the passage number, respectively. Numbers indicate the location of RNA segments. Arrows indicate rearranged segments 7R, 7RΔ, and 11R.

To assess whether r-RV could overgrow wt-RV or rearranged segments could segregate preferentially to wt segments in viral progenies, competitive experiments were performed between r-RV (M1 to M4) and a wt-RV having an entirely different genetic background. The RV bovine strain RF (RF virus) was chosen to be used as wt-RV because of its clear growth advantage over human RV, growing to titers 100 to 1000 fold higher than those of human wt or r-RV M0-M4 (see Figure 2) and because it could easily be distinguished from human r-RV based on RNA profiles (Figure 5). Competitive experiments were first performed by co-infecting MA-104 cells at the same MOI (0.3 PFU/cell) by the RF virus and either of the human r-RV (M1–M4) (Figure 5). The RNA profile of the viral inoculums used for each mixed infection was determined as a control, showing the superimposition of RNA segments from both bovine and human viruses. In all cases, rearranged segments segregated in viral progenies produced during competitive experiments. Indeed, viral progenies obtained after the first passage in MA-104 cell culture, consisted of reassortant viruses that had inherited rearranged segments 7 (Figure 5A, B), 11 (Figure 5C), or both (Figure 5D) from human r-RV, whereas other RNA segments that could be discriminated by PAGE were derived from the bovine RF virus. During further passages, rearranged segments were maintained in the reassortant viral progenies. Additionally, some segments from human r-RV could also be faintly visible on the RNA profiles indicating that the viral progeny possibly included several other reassortant viruses.

**Figure 6:**
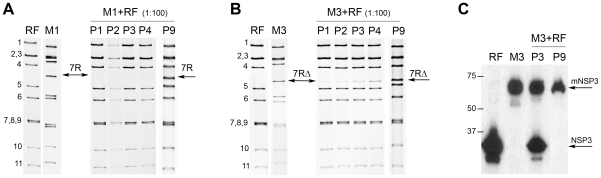
Competitive experiments between human r-RV and bovine wt-RV at an MOI ratio of 1∶100. Competitive experiments were performed by co-infecting MA-104 cells by 0.003 PFU/cell of r-RV M1 or M3 and 0.3 PFU/cell of bovine wt-RV RF (1∶100 MOI ratio). RNA profiles of viral progenies resulting from mixed infections by wt-bovine RF virus and human r-RV M1 (A) or M3 (B). Pn indicate the passage number; numbers indicate the location of RNA segments; arrows indicate rearranged segments 7R and 7RΔ. (C) Western-blot detection of the NSP3 protein expressed by the viral progenies resulting from the M3+RF co-infection (1∶100 MOI ratio) at passage 3 (P3) and 9 (P9). Arrows indicate the NSP3 and the modified mNSP3 proteins expressed by wt-RV RF and r-RV M3, respectively. Numbers indicate molecular size, in kilodaltons.

To assess whether rearranged segments actually segregated preferentially to other wt RNA segments, competitive experiments were then performed using an MOI ratio of one human r-RV (M1 or M3) to 100 bovine RF virus for the initial mixed infection. Under these conditions, the resulting viral progenies had RNA profiles matching RF virus, except for the gradual appearance of a rearranged segment 7 (7R or 7RΔ for mixed infection with M1 or M3, respectively), which was only faintly visible at the first passages and increased in intensity during subsequent passages (Figure 6). At passage 9, rearranged segments 7R or 7RΔ were detected in an equimolar ratio to other RNA segments indicating that they had most probably replaced the bovine wt segment 7, although this could not be ascertained on the RNA profile because wt segments 7, 8 and 9 of RF virus co-migrated as a triplet that could not be easily resolved. However, for competition between RF and M3 viruses, replacement of RF wt segment 7 encoding the NSP3 protein with M3 rearranged segment 7RΔ encoding the mNSP3 protein could be established using Western-blot analysis. Indeed, while both NSP3 and mNSP3 proteins were detected at passage 3, only mNSP3 was detected at passage 9.

Taken together these results indicated that rearranged RNA segments segregated preferentially to their wt heterologous counterparts in reassortant viral progenies resulting from mixed infection by two RV with heterologous genetic backgrounds. This raised the question whether this preferential segregation could be due to a better incorporation of rearranged segments into viruses, solely related to their rearranged structure, or to a growth advantage conferred to reassortant viruses by the heterologous genes, irrespective of their rearranged structure.

### Selection of rearranged over wt segments in reassortant viruses is not related to a growth advantage

Bovine reassortant viruses were isolated after 3 plaque-to-plaque purification steps from viral progenies obtained at passage 9 after mixed infection between RF and M1 or M3 viruses (see Figure 6). Reassortant viruses RF7R and RF7RΔ had inherited rearranged segment 7R and 7RΔ from M1 and M3 human viruses, respectively, in the genetic background of RF virus.

**Figure 7:**
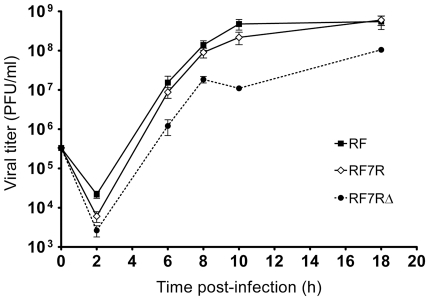
One-step viral growth curves of bovine reassortant viruses RF7R and RF7RΔ. For each virus (RF, RF7R and RF7RΔ), confluent MA-104 cells were inoculated at the same MOI of 0.5 PFU/cell. The infected cell cultures were harvested at 2, 6, 8, 10, and 18 hours post-infection and virus titers were determined by plaque assay in MA-104 cells. The rearranged segment 7 of bovine reassortant viruses RF7R and RF7RΔ derives from human r-RV M1 and M3, respectively.

**Figure 8:**
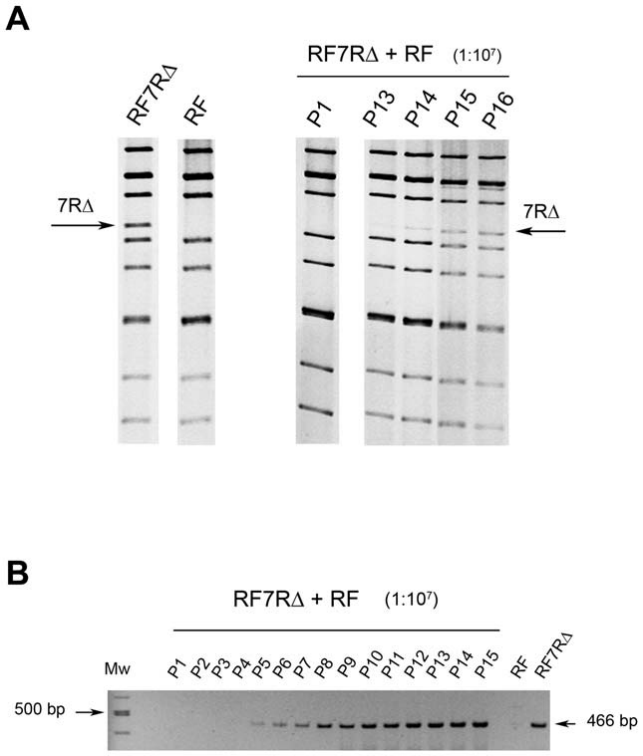
Competitive experiments between reassortant RF7RΔ and wt-RF viruses at an MOI ratio of 1∶10^7^. Competitive experiments were performed by co-infecting MA-104 cells by 3×10^−7^ PFU/cell of RF7RΔ and 3 PFU/cell of wt-RV RF (1∶10^7^ MOI ratio). The resulting viral progeny was serially propagated in MA-104 cells and viral dsRNA was analyzed by PAGE (A) and RT-PCR for specific detection of rearranged segment 7 (B). Pn indicates the passage number; arrows indicate the location of segment 7RΔ and the expected size of the PCR product (466 bp).

To assess whether rearranged segments 7 could have conferred a growth advantage to the bovine reassortant viruses, growth curves of RF, RF7R and RF7RΔ viruses were established in MA-104 cell culture over an 18 hours period of infection. The growth kinetics of reassortant RF7R and RF viruses were quite similar, and reassortant RF7RΔ grew to titers 10 fold lower than those of RF virus (Figure 7). Reassortant virus RF7RΔ was in turn used for competitive experiments with wt RF virus. Ten fold serial dilutions of RF7RΔ (MOI ranging from 3 to 3×10^−7^ PFU/cell) were combined with a constant amount of wt-RV RF (MOI of 3 PFU/cell) and used for primary inoculums (MOI ratio of reassortant to wt virus ranging from 1∶1 to 1∶10^7^). During further serial passaging in cell culture, the rearranged segment 7RΔ was always detected by PAGE in the resulting viral progenies, although after a number of passages that was related to the initial ratio of reassortant to wt virus (at passage 1, 3, 5, 7, 10, 12, 13 and 15, for ten fold serial ratios of 1∶1 to 1∶10^7^, respectively). Results obtained using a ratio of 1∶10^7^ are shown in figure 8A. Using an RT-PCR assay designed to specifically detect low copy number of rearranged segments 7 among a vast majority of their wt counterparts, the rearranged segment 7RΔ became detectable in the viral progeny after only 5 passages, and its amount increased during subsequent passages, as judged by the intensity of the PCR signals (Figure 8B). Similar results were obtained from competitive experiments with reassortant RF7R and wt RF virus. Given that the substitution of wt by rearranged segment 7 did not result in any growth advantage for the virus, the constant occurrence of this substitution in the viral progenies resulting from mixed infections, strongly suggested that preferential segregation of rearranged RNA segments could be related to a mechanism of preferential packaging of rearranged segments into viruses.

## Discussion

Rotaviruses carrying rearranged segment(s) (r-RV) can package up to 10% additional bp without morphological modification [34] nor being defective, since r-RV can be isolated by limit dilution or plaque methods [11], [12], [16], [29], [31]. However, the impact of gene rearrangement on viral replication efficiency is not fully understood.

In this study, results obtained from one-step viral growth curves indicate that human r-RV replicate less than or equally to their normal counterpart. Prior studies have reported similar results for bovine or porcine RV carrying rearranged segment(s) [11], [19], [30], [31]. In a cell-free RV replication system, Patton *et al.* have shown that a size increase of the RNA template has a strong negative influence on dsRNA synthesis by open cores. However, the 1.5-fold size increase of a rearranged RNA segment does not affect its replication efficiency [35]. Thus, findings reported from cell-free system are in agreement with those observed in cell culture, indicating that the size increase of rearranged RNA segments does not affect the viral growth. In viral progenies resulting from competition between human r-RV and homologous wt-RV, rearranged segments 7, 11, or both, were always found to substitute their wt counterpart. For competitive experiments, we have chosen to perform serial passages in cell culture at high MOI (undiluted inoculum) to favor co-infection of cells by both r-RV and wt-RV. Under these conditions, our findings are in agreement with those of previous studies that report the selection of r-RV over wt-RV in the course of mixed infection for a bovine r-RV (strain brvA) with a rearranged segment 5 [11], a porcine r-RV (strain CC86) with a rearranged segment 11 [30], and a reassortant human r-RV (strain C11) carrying a bovine rearranged segment 6 [31]. Taken together, lack of growth advantage conferred by rearranged segments to r-RV, combined with the constant selection of rearranged segments in viral progenies, strongly suggests a preferential segregation of rearranged segments rather than an overgrow of r-RV over wt-RV during viral replication.

We then wondered whether preferential segregation of rearranged segments in viral progenies that we observed for r-RV and wt-RV with homologous genetic backgrounds, could also occur for r-RV and wt-RV with different genetic backgrounds. This could be hypothesized, since several studies have reported the possibility of obtaining viable reassortant viruses that have inherited a rearranged segment derived from a different genetic background [29], [31], [36], [37]. By performing competitive experiments between human r-RV and the bovine wt-RV strain RF, chosen for its major growth advantage over human RV, we show here that rearranged segments 7 and/or 11 of the human RV also underwent preferential segregation in the heterologous genetic background of the bovine RV, replacing the wt segment in the viral progenies. A dynamic progressive selection of the rearranged segment in a heterologous genetic background throughout passages was indeed observed by PAGE, and still occurred – but at later passages – when disadvantageous proportions of r-RV to wt-RV were used for the initial inoculum.

The preferential segregation of rearranged segments could be the result of either a better incorporation into viruses due to their intrinsic nature, or of a growth advantage conferred to reassortant viruses by the heterologous genes, irrespective of their rearranged structure. Co-infections (at 1∶100 MOI ratio) between r-RV M1 or M3 (carrying the same wt segment 11 as M0) and bovine RV RF showed that wt segments 11 of human viruses were not selected in the resulting viral progenies (Figure 6A and 6B). In the same way, co-infections (at 1∶100 MOI ratio) between M4 (carrying the same wt segment 7 as M0) and RF showed that wt segment 7 of the human virus was not selected in the viral progeny; similarly, mixed infections between wt-RV M0 (wt segments 7 and 11) and the bovine always resulted in the selection of viruses with a RF RNA profile (results not shown). Thus, unlike rearranged segments, wt segments 7 and 11 of human RV are not selected in the viral progenies resulting from mixed infections with the bovine RV. This rules out the possibility that preferential segregation of rearranged segments 7 and 11 in the bovine RV could have occurred only because these segments were originated from human RV. Moreover, a selective advantage resulting from the substitution of NSP3 or NSP5/NSP6 proteins of bovine RV by the corresponding human RV proteins is unlikely. Indeed the replacement of the bovine-RV NSP3 gene by the human-RV NSP3 gene did not affect growth efficiency, since the wt-RV RF and the r-RV bovine reassortant RF7R (carrying the human rearranged segment 7R in the bovine RV background) had similar growth kinetics. Moreover, replacement of the wt bovine NSP3 protein by the modified mNSP3 protein (RF7RΔ) decreased viral growth efficiency. The constant selection of RF7R and RF7RΔ in the progeny during competition with wt-RF strongly supports the hypothesis of a preferential segregation only based on the intrinsic nature of rearranged segments, and which is independent of the genetic background.

In the light of our results, the most plausible explanation for the preferential segregation of rearranged segments emerge as a selective advantage of rearranged RNA segments to be encapsidated into virions, as evoked in the literature [30]. This mechanism would be efficient enough for consistently selecting the rearranged segment among a large majority of its wt counterpart, although not conferring any growth advantage to the resulting reassortant viruses. When the initial inoculum contains unbalanced proportions of r-RV to wt-RV, co-infection of the same cell by both viruses should be a rare event. Even if rare, co-infection events will contribute to the enrichment of the r-RV population in the viral progeny, which, in turn, will increase the frequency of co-infections, leading to the progressive expansion of r-RV over wt-RV during subsequent cell passages. Indeed, we found that the number of passages required for rearranged segments to replace wt-segments increased inversely to the ratio of r-RV to wt-RV used for the initial inoculum. We thus consider the number of passages required for the expansion of rearranged segments in the viral progeny as a direct indicator of the packaging efficiency rate of rearranged segments. Considering that when starting with 10-fold serial ratios of r-RV to wt-RV ranging from 1∶1 to 1∶10^7^, the rearranged segment was detectable by PAGE at passage 1, 3, 5, 7, 10, 12, 13 and 15, respectively, and that PAGE detection is indicative of a rearranged segment to wt segment ratio ≥1∶1, one can estimate that the proportion of rearranged segments to wt segments increases approximately by 10-fold every 2 or 3 passages, i.e. by 2.15- to 3.15-fold at each passage. By comparison, Xu *et al.* have described that after a mixed infection by a 1∶1 ratio of the human RV strain Wa and the r-RV reassortant C11 (carrying a bovine rearranged segment 6 in the background of Wa), the viral progeny at passage 1 comprised 85% of r-RV [31], which can be calculated as a 1.7-fold increase during this passage.

Our results combined with data from the literature support the hypothesis of preferential packaging as a common property of rearranged RNA segments to explain the selection of r-RV over wt-RV. However, the reasons why rearranged segments are preferentially packaged into RV remain to be determined. It could be hypothesized, like suggested previously for genotypic variants of orbivirus [38], that duplication of packaging signals or secondary structures in rearranged segments may increase their probability to be encapsidated. Packaging signals remain to be identified for RV. Concerning reovirus and bluetongue virus (BTV), two other *Reoviridae* viruses, packaging signals have been identified with the help of reverse genetics systems, and exceed the 5′- and 3′- UTR over the coding sequences [39]–[43]. Sequence comparison between RV strains has contributed recently to identify conserved sequences and/or secondary structures in the RV genome [44], among which some are probably involved in packaging. It would be of interest to perform a similar comparison for r-RV strains to identify conserved sequences and/or secondary structures that are duplicated in the rearranged segments. For rearranged genes that were used in this study, parts of the 5′ sequences are duplicated, while the 3′-untranslated terminus is unique, which can indicate that, as for reovirus and BTV, the packaging signals might be located in the 5′ region and include coding sequences.

Paradoxically, if rearranged RNA segments have a selective advantage in packaging, why all RV do not possess rearranged segment(s)? First of all, viral replication and selection of viral populations might be different in vivo and under cell-culture conditions, since host cell factors could have a selective effect on segregation of rearranged segments as suggested by Graham *et al.* [37]. Next, although rearrangement events can be detected during acute infection in immunocompetent children, r-RV remain clearly in a minority compared to wt-RV in the viral population [20]. Since RV acute infection is of short duration and constrained by the immune response, the number of viral replication cycles at high MOI might be insufficient for the emergence of the r-RV over the wt-RV population. Conversely, r-RV are constantly recovered in the course of chronic RV infection of immunocompromised children [5], [6], [16], with a dynamic kinetic over time that is consistent with the numerous passages required *in vitro* for expansion of r-RV when initially present in a minority.

Even if, as compared to gene reassortment, gene rearrangement does not represent a significant mechanism in generating genetic diversity, it can offer a tool for a better understanding of RV biology. Indeed, our results bring some light on a specific property of rearranged segments over which was based the reverse genetics system for RV that we described recently [32]. This system allowed the rescue, with no other selection pressure than serial passages in cell culture, of recombinant viruses carrying cDNA-derived rearranged segments 7, including an infectious virus expressing a modified recombinant NSP3 protein.
